# A Deep Reinforcement Learning-Based MPPT Control for PV Systems under Partial Shading Condition

**DOI:** 10.3390/s20113039

**Published:** 2020-05-27

**Authors:** Bao Chau Phan, Ying-Chih Lai, Chin E. Lin

**Affiliations:** 1 Department of Aeronautics and Aeronautics, National Cheng Kung University, Tainan 701, Taiwan; pbchau.hk09@gmail.com (B.C.P.); chinelin@mail.ncku.edu.tw.com (C.E.L.); 2 UAV Center, Chang Jung Christian University, Tainan 701, Taiwan

**Keywords:** solar PV, maximum power point tracking (MPPT), partial shading condition (PSC), deep Q network (DQN), deep deterministic policy gradient (DDPG)

## Abstract

On the issues of global environment protection, the renewable energy systems have been widely considered. The photovoltaic (PV) system converts solar power into electricity and significantly reduces the consumption of fossil fuels from environment pollution. Besides introducing new materials for the solar cells to improve the energy conversion efficiency, the maximum power point tracking (MPPT) algorithms have been developed to ensure the efficient operation of PV systems at the maximum power point (MPP) under various weather conditions. The integration of reinforcement learning and deep learning, named deep reinforcement learning (DRL), is proposed in this paper as a future tool to deal with the optimization control problems. Following the success of deep reinforcement learning (DRL) in several fields, the deep Q network (DQN) and deep deterministic policy gradient (DDPG) are proposed to harvest the MPP in PV systems, especially under a partial shading condition (PSC). Different from the reinforcement learning (RL)-based method, which is only operated with discrete state and action spaces, the methods adopted in this paper are used to deal with continuous state spaces. In this study, DQN solves the problem with discrete action spaces, while DDPG handles the continuous action spaces. The proposed methods are simulated in MATLAB/Simulink for feasibility analysis. Further tests under various input conditions with comparisons to the classical Perturb and observe (P&O) MPPT method are carried out for validation. Based on the simulation results in this study, the performance of the proposed methods is outstanding and efficient, showing its potential for further applications.

## 1. Introduction

Energy demand has been continuously increasing and is predicted to rise at a significant rate in the future [1]. It leads to the rapid development of renewable energy resources like solar, wind, tidal, geothermal, etc., for reducing the consumption of fossil fuels and protecting the global environment from pollution. Besides wind power, solar energy is the most commonly used energy source with a high energy market share in the energy industry around the world [2]. Due to the continuous decline in price and the increasing concern of greenhouse gas emissions, lots of photovoltaic (PV) systems have been intensively constructed, especially in areas with rich solar radiation.

Besides the efforts of improving the production process of the PV module and converter power electronics for better performance of the system, it is essential to enhance the system throughput with an efficient maximum power point tracking (MPPT) controller. The MPPT algorithm is employed in conjunction with a DC/DC converter or inverter to assure the MPP can always achieve the goal under different weather conditions of solar radiation and temperature. Over the years, numerous MPPT methods have been employed, which can be classified into various categories according to sensor requirements, robustness, response speed, effectiveness, and memory as shown in these review papers [2,3,4]. The conventional MPPT methods [5] that have been practically adopted due to their simplicity and easy implementation. In which, Perturbation and Observation (P&O) and Incremental Conductance (IC) are the famous algorithms. Moreover, many other traditional algorithms have been introduced by Karami [6], such as Open Circuit Voltage (OV), Ripple Correlation Control (CC), Short Circuit Current (SC), One-Cycle Control (OCC). Mohapatra [7] has confirmed that conventional methods can usually perform efficiently under a uniform solar radiation condition. However, being trapped at a local MPP resulting in low energy conversion under a partial shading condition (PSC) is their considerable drawback. In addition, a small step size of the duty cycle causes longer tracking time, while it can oscillate around the MPP with the large one. Ahmed [8] tried to modify the P&O method with variable step size to eliminate its drawbacks of slow tracking speed, weak convergence, and high oscillation. In this scenario, the controller can choose a large step size when the MPP is still far away. As it approaches the MPP, the small step size is used to reduce the oscillation. Other modified methods can be found in [2,3,4,5].

Another class of MPPT control is based on soft computing techniques as summarized by Rezk [4], such as fuzzy logic control (FLC) [9], artificial neural network (ANN) [10], and neuro-fuzzy (ANFIS) [11,12]. While some methods are proposed based on the evolution algorithms, like genetic algorithm (GA) [13], cuckoo search (CS) [14], ant colony optimization (ACO) [15], bee colony algorithm (BCA) [16], bat-inspired optimization (BAT) [17], bio-inspired memetic salp swarm algorithm [18], etc. Jiang [19] has defined that these methods, based on both soft computing techniques and evolutionary algorithms, can efficiently deal with the nonlinear problem and obtain global solutions or are able to track the global MPP under PSCs. However, they have two significant disadvantages. It generally requires an expensive microprocessor for less computational time and the knowledge of a specific PV system for low convergence randomness. Rezk et al. [4] have shown that the method based on particle swarm optimization (PSO) is currently popular in the application of MPPT control [20]. It can uniquely combine with other algorithms to create a new approach for efficiently solving the MPPT control problems, such as PSO with P&O by Suryavanshi [21], and PSO with GA by Garg [22], etc.

Recently, extensive studies have focused on reinforcement learning (RL) with various successful applications due to its superior learning ability from environmental-interacting historical data, instead of the requirement of complex mathematical models of the control system in conventional approaches [23,24]. As summarized by Kofinas et al. [25], RL has higher convergence stability with shorter computational time compared to meta-heuristic methods, thus making it a potential tool for optimally solving the problem of MPPT control. To date, a few studies have focused on this field, in which Q-learning is the most-used algorithm. In [26], Wei has applied MPPT control for a variable-speed wind energy system based on Q-learning. The authors in [27] also developed an MPPT controller for a tidal energy conversion system. Additionally, the works that try to implement RL for the MPPT control of a solar energy conversion system can be found in [25,28,29]. However, these approaches have the drawbacks of low state and action spaces. Kofinas et al. [25] have used a combination of 800 states with five actions to form a state action space of 4000 state actions, while Hsu et al. [28] and Youssef [29] just made only four states. As a consequence, the system with large state and action spaces results in longer computational time. Phan and Lai [30] proposed a combination of Q-learning and P&O methods. Each control area, which is divided based on the temperature and solar radiation, are handled by a Q-learning controller for learning the optimal duty cycle. Then, these optimal duty cycles are forward to the P&O controller resulting in the smaller step size used. Chou [31] has developed two MPPT algorithms based on RL, one uses a Q table and the other one adopts a Q network. However, the problems under PSCs are not mentioned in the above studies. Instead of using a trained agent, the approaches [32,33] deal with the MPPT control problem by using multiple agents. A novel memetic reinforcement learning-based MPPT control for PV systems under partial shading condition was developed [32] while a transfer reinforcement learning approach was studied to deal with the problem of global maximum power point tracking [33]. Generally, the major drawback of the methods, as mentioned above, is the use of small discrete state and action space.

The recent development of machine learning leads to an integration of reinforcement learning and deep learning, named as deep reinforcement learning (DRL), which is considered as a powerful and potential tool to deal with the optimization control problem [34,35,36]. The successful performance of the DRL method in playing Atari and Go games is described in the study [37]. DRL is a powerful method for handling complex control problems with large state spaces. The advantage of DRL is that it can manage the problem with continuous state and action spaces. To date, DRL has been successfully applied to several fields, including games [37], robotics [35,38], natural language processing [39], computer vision [38], healthcare [40], smart grid [41], etc. Zhang [42] has defined a brief overview of DRL for the power system. A similar concept with deep reinforcement learning has been developed for MPPT control of the wind energy conversion system, in which a neural network is used as a function approximation to replace the Q-value table [43,44].

After an exhaustive search of related works and the achievement of reinforcement learning (RL), it is shown that there is a gap in the application of the DRL algorithm for MPPT control. Therefore, this paper proposes MPPT controllers based on DRL algorithms to harvest the maximum power and improve the efficient and robust operation of the PV energy conversion systems. In this study, two model-free DRL algorithms, including deep Q network (DQN) and deep deterministic policy gradient (DDPG), are introduced to the MPPT controllers. Different from the RL-based method, which can only operate with discrete state and action spaces, both proposed methods can deal with continuous state spaces. In which, DQN works with discrete action space; while the continuous action space is used in the DDPG method. Rather than using a lookup table to store and learn all possible states and their values in the RL-based method, which is impossible with large discrete state and action spaces, the DRL-based method uses neural networks to approximate a value function or a policy function. The main contributions of this paper are as follows:

- Two proposed efficient and robust MPPT controllers for PV systems based on DRL are proposed and simulated in MATLAB/Simulink, including DQN and DDPG.
- Eight scenarios under different weather conditions are considered for testing the performances of the two proposed methods. They are divided into four scenarios under uniform conditions and four other scenarios under partial shading conditions, as shown in Table 3.
- A comparison between the proposed method and the P&O method is also investigated.

In this paper, the descriptions of a PV mathematical model and the influence of partial shading conditions to the location of MPP are introduced in Section 2. The proposed methods based on two different reinforcement learning algorithms, including DQN and DDPG, are described and formulated in Section 3. Based on the simulation and the comparison results in Section 4, the performance of the proposed methods appears very outstanding and efficient in PV operation. Finally, the conclusion and future work are presented in Section 5. 

## 2. Modelling of PV Module under PSC

### 2.1. Mathematical Model of PV Module

PV solar cells generally have a p–n junction which is fabricated in a thin layer of semiconductor materials to convert the solar irradiation into electricity [30]. It is important to employ a reliable solar cell model to simulate a PV system. There is a trade-off between desirably accurate models and computing speed. There are two types of PV models, including double-diode and single-diode [6]. Although a single-diode model is less accurate than the other one, it is preferred due it its simplicity. A solar cell equivalent electrical circuit of a single-diode model is used in this study [28]. Based on Kirchhoff’s law, the output current of an ideal cell is given by [3,28,45]:

(1) $$I=I_{ph}-I_{d}-I_{sh}$$

where $I_{sh}$ is the parallel resistance current, which is given by

(2) $$I_{sh}=\frac{V+IR_{s}}{R_{p}}$$

where $R_{s}$ is the series resistance because of all the components that come in the path of the current which is desired as low as possible, and $R_{p}$ is parallel resistance which is desired as high as possible. 

Additionally, $I_{ph}$ is the light generated current, which is proportional to the light intensity. It is calculated by

(3) $$I_{ph}=[I_{sc}+K_{I}(T_{c}-T_{r})]\times \frac{G}{G_{STC}}$$

where $I_{sc}$ is the current of short circuit at standard testing condition (STC) ($T=25\;℃,\;\;G_{STC}=1000\;\;W/m^{2}$) and $K_{I}$ is the cell short-circuit current temperature coefficient. $T_{c}$ is the cell operating temperature, while $T_{r}$ is the reference temperature, and G is the relative irradiation. $I_{d}$ is the diode current, which is given by

(4) $$I_{d}=I_{0}\left[\exp\left(\frac{qV_{d}}{AkT_{c}}\right)-1\right]$$

where $q=1.6\times 10^{-19}$ is the electronic charge, $k=1.38\times 10^{-23}$ is the Boltzmann’s constant, and A is the ideal factor of the diode. $I_{0}$ is the reverse saturation current of the diode, while $V_{d}$ is the voltage of the equivalent diode. They are calculated by

(5) $$V_{d}=V+IR_{s}$$

PV cells are usually connected in series to become a PV module. A simple mathematical model for calculating the current of a PV module, which is simultaneously dependent on the solar irradiation and temperature, is given by

(6) $$I_{pv}=I_{ph}-I_{O}\left[\exp\left(\frac{q\left(V+IR_{s}\right)}{AkT_{c}N_{s}}\right)-1\right]$$

where $N_{s}$ is the number of series resistance cells. 

As described in the equation above, the characteristics of a PV module are heavily affected by environmental factors. In this study, the American Choice Solar ACS-335-M PV module is used for the simulation of a PV system. Its specification is illustrated in Table 1. Supplementary Figure S1 illustrates the current–voltage (I–V) and power–voltage (P–V) curves of the PV module with different irradiations under the same temperature. As the irradiation rises, the curve moves downwards with the reduction of the maximum power point value. In addition, the plots of I–V and P–V curves under several temperatures with constant irradiation at 1000 W/m^2^ are provided in Supplementary Figure S2. It is clearly shown that there is a decline in the power caused by the escalation of temperature.

### 2.2. Partial Shading System Effect

A PV array consists of several PV modules, connected in series or parallel to get the desired output voltage and current. Two PV modules in series mean that there are a maximum two peaks along the P–V curve under PSC. Similarly, five PV modules in series could have a maximum five peaks. The proposed method in this study can be applied for different PV systems. However, for the simplicity and clear distinction between a global maximum power point (MPP) and local MPPs, three PV modules in series are used for the simulation. Supplementary Figure S3 shows the PV array used for the simulation in this study. As shown in the diagram, bypass diodes and a blocking diode are used to protect PV modules from self-heating under partial shading conditions (PSCs) [2,3]. Here, if more than one PV module is shaded by pole shadows, building shadows, and bird droppings, it causes the partial shading over a PV string. Here, it acts as a load rather than a power source. The hot spots phenomenon will damage the shaded PV module in long term conditions [14,46,47]. Hence, a bypass diode is added in parallel to protect the PV system and eliminates thermal stress happening on PV modules.

Under uniform solar irradiation, the bypass diode is reverse biased. It is forward biased when a PV module is shaded, and the current passes through the diode instead of the PV module. However, with a bypass diode, the condition of partial shading causes multiple peaks on the power curve, including local and global maxima. If the system is operated at the global maximum power point (GMPP) to extract the maximum energy from the PV array, up to 70% of power loss could be eliminated [2]. Supplementary Figure S4 shows the power curves under uniform and partial shading conditions. It leads to a conclusion that an intelligent and efficient MPPT method should be used under PSCs to distinguish between a global MPP and local MPPs. Conventional MPPT algorithms, such as P&O and IC, usually stop searching when they reach the first peak, so it is unable to distinguish between global and local MPPs. Hence, in this paper MPPT controllers based on DRL algorithms are proposed and tested with different input conditions to ensure the GMPP is achieved at all times.

### 2.3. PV System Introduction

PV solar has nonlinear characteristics, where its performance is significantly affected by the change of temperature and solar irradiance. It is clear from the previous figures that the PV output power is directly proportional to the decline of solar irradiance and inversely proportional to the temperature. This means that only one optimum terminal voltage of the PV array exists, which lets the PV panel operate at the MPP with a specific weather condition [47,48]. Thus, it is important to develop a robust MPPT control for extracting the MPP at all times [7]. In addition, under PSCs, there are multiple peaks on the P–V curve of a PV panel. Hence, a smart MPPT controller should be considered to overcome the limitation of traditional MPPT methods. 

A block diagram of a PV system is demonstrated in Supplementary Figure S5, including a PV array, a DC–DC converter, a resistance load, an MPPT controller. Here, DC–DC converters have a major role in the MPPT process. When connecting output terminals of a PV array with a DC–DC converter, the array voltage can be controlled by changing the duty cycle D, which is a pulse width modulation (PWM) signal and is executed by the MPPT controller to regulate the voltage at which maximum power is obtained. The calculation of the duty cycle for a DC–DC boost converter is given by [30]

(7) $$D=1-\frac{V_{in}}{V_{out}}$$

In this paper, two deep reinforcement learning algorithms are applied for MPPT control, including DQN and DDPG. The principles of these two algorithms, applied for MPPT control of a PV system, are introduced in the next section.

## 3. Deep Reinforcement Learning based MPPT Control

### 3.1. Basic Concept of DRL

As DRL can be considered as an advanced reinforcement learning (RL), a brief introduction of RL is firstly given below. RL is a class of unsupervised machine learning methods, which are derived from neutral stimulus and response between the agent and its interacting environment [49]. With the recent development of the computer science industry, reinforcement learning has become more popular in solving sequential decision-making problems [24,36,50]. RL is applied to figure out a policy or behavior strategy, that maximizes the total expected discounted rewards by trial-and-error interaction with a given environment [51]. The general model of RL includes an agent, an environment, actions, states, and rewards [23]. Then, the environment represents the object that the agent is acting on, while the agent refers to the RL algorithm. The environment starts to send a state, based on its knowledge the agent will take an action in response to that state. Then, it receives a pair of next state and reward from the environment. After that, the agent will update its knowledge with the reward to evaluate its last action. When the environment sends a terminal state, the episode ends and the other one will begin. The loop keeps going on until the designed criteria are met [23].

To find an optimal policy, some algorithms use the value function $V^{\pi }\left(s\right)$, which defines how good it is for the agent to reach a given state [51]. It is the expected return when following policy π from the state s. In addition, some other methods are based on the action-value function $Q^{\pi }\left(s,a\right)$, which represents the expected return of taking this action a in the current state s under a policy π. The $V^{\pi }\left(s\right)$ and $Q^{\pi }\left(s,a\right)$ functions are calculated as below [23,42,51]:

(8) $$V^{\pi }\left(s_{t}\right)=Ε\left[R_{t}|s_{t}=s\right]=Ε\left[\sum_{k=0}^{\infty }\gamma^{k}r_{t+k+1}|s_{t}=s\right]$$

(9) $$Q^{\pi }\left(s_{t},a_{t}\right)=Ε\left[R_{t}|s_{t}=s,a_{t}=a\right]=Ε\left[\sum_{k=0}^{\infty }\gamma^{k}r_{t+k+1}|s_{t}=s,a_{t}=a\right]$$

Q-Learning is an off-policy, model-free RL algorithm, which has been increasingly popular in various fields. In Q-Learning, the $Q^{\pi }\left(s,a\right)$ function can be presented as an iterative form by the Bellman equation as below [23,51]:

(10) $$Q^{\pi }\left(s_{t},a_{t}\right)=Ε\left[r_{t+1}+\gamma Q^{\pi }\left(s_{t+1},a_{t+1}\right)|s_{t},a_{t}\right]$$

Over the long run, the maximum cumulative reward is achieved by an optimal policy $\pi^{*}$. At this time the best value function and action-value function are given by [23]

(11) $$\pi^{*}=\underset{\pi }{\arg\max V^{\pi }}\left(s\right)$$

(12) $$V^{*}\left(s\right)=\underset{\pi }{\max V^{\pi }}\left(s\right)$$

(13) $$Q^{*}\left(s,a\right)=\underset{\pi }{{\max Q}^{\pi }}\left(s,a\right)$$

One of the most interesting areas of AI today is the deep reinforcement learning (DRL) algorithm, where an agent can learn on its own based upon the interacting results with a specific environment. DRL, which is the combination of RL and deep learning, has significantly achieved great success in various fields, such as robotics, games, natural language processing, and the management of finance and business. One of the major disadvantages of RL is using a look-up table to store, index, which is sometimes impossible for real-world problems with large state-and-action spaces. Hence, a neural network can be adopted to approximate a value function, or a policy function [37,51]. That is, neural nets can learn how to map states or state-action pairs to Q values.

**Figure 1:**
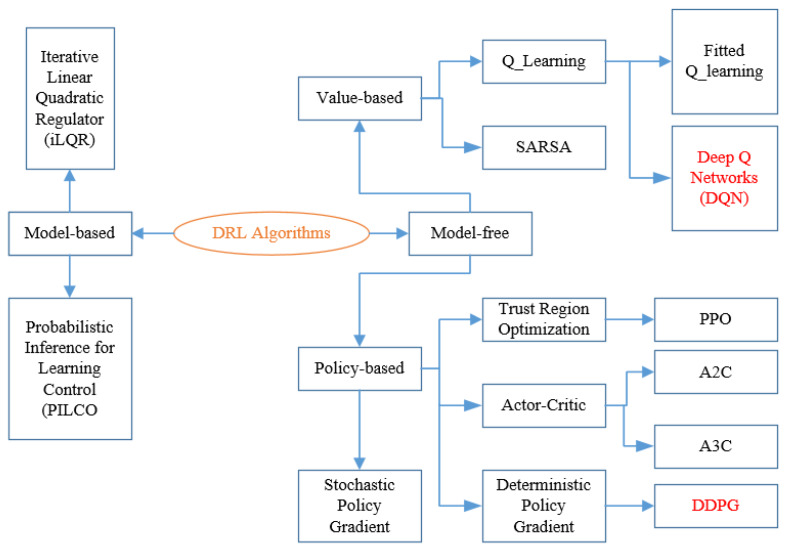
Introduction of deep reinforcement learning (DRL) algorithms.

As shown in Figure 1, there are two types of solution methods, including model-based, model-free. In model-based DRL, the model is known or learned. The strong advantage of the model-based method is that it requires few samples to learn. However, it is far more computationally complex when the model becomes surprisingly tricky to learn. On the other hand, model-free RL will be more favorable to deal with. No accurate representation of the environment to be effective is needed and it is also less computationally complex. In model-free DRL, it is divided into value-based and policy-based. Value-based try to improve the value function every iteration until reaching the convergence condition. Here, the objective function and updating method are given below [36,42]:

(14) $$J\left(\theta \right)=Ε\left[{\left(r_{t+1}+\gamma \underset{a}{max}Q\left(s_{t+1},a_{t+1}|\theta \right)-Q\left(s_{t},a_{t},|\theta \right)\right)}^{2}\right]$$

(15) $$\theta_{t+1}=\theta_{t}+\alpha \left(r_{t+1}+\gamma \underset{a}{max}Q\left(s_{t+1},a|\theta \right)-Q\left(s_{t},a_{t}|\theta \right)\right)\nabla_{\theta }Q\left(s_{t},a_{t}|\theta \right)$$

where α is learning rate, and θ is the weights of the neural network. 

In the policy-based methods, the quantity of interest is directly optimized by updating the policy at each time step and computing the value based on this new policy until getting the policy convergence. Firstly, the gradient of the objective function is defined and the weight matrix will be updated as below [36,42]:

(16) $$\nabla_{\theta }J\left(\theta \right)=Ε\left[\sum_{t=0}^{T}\nabla_{\theta }\log\pi_{\theta }\left(a_{t}|s_{t}\right)\sum_{t=0}^{T}r\left(s_{t},a_{t}\right)\right]$$

(17) $$\theta \leftarrow \theta +\alpha \nabla_{\theta }J\left(\theta \right)$$

### 3.2. Markov Decision Process Model of a PV System

To implement an RL or DRL approach on MPPT control of a PV system, a Markov Decision Process (MDP) model of the PV system behavior needs to be defined. Almost all RL problems can be considered as MDPs. Before starting a detailed description of the deep reinforcement learning, this part provides short background information on the concept of MDP models applied for the MPPT control problem.

Formally, an MDP is considered as a tuple S, A, T, R. S is a finite set of states which describes the all the operating point of the PV system, while R is a finite set of actions, which is the perturbation of the duty cycle and is applied on the converter to change the state of operation of the PV source. T is the transition function, while R is the reward function, representing how much immediate reward we expect to get at the moment when an action is performed from a current state. They are given by [23]

(18) $$P_{ss^{\prime }}^{a}=Ρ\left[S_{t+1}=s^{\prime }|S_{t}=s,A_{t}=a\right]$$

(19) $$R_{s}^{a}=Ε\left[R_{t+1}|S_{t}=s,A_{t}=a\right]$$

The agent learns how to obtain the maximum total reward getting over an episode develop a strategy or a policy. Thus, we reinforce the agent with positive rewards for choosing a correct action with good performances, as well as negatives rewards for poor performances [23]. For the implementation of RL and DRL on an MPPT control, the calculations of predefined state and action spaces, as well as the reward, are defined. The observation is represented by the combination of voltage, current, duty cycle, and its perturbation as below [25,28]:

(20) $$S=\{V_{pv},I_{pv},D,\Delta D\}$$

Action-spaces are the perturbations of duty cycle ∆*D*, including negative, positive, and no change:

(21) A={a|+ΔD,0,−ΔD}

Reward function is defined as below:

(22) $$r=r_{1}+r_{2}+r_{3}$$

(23) $$r_{1}=\frac{P_{t+1}}{P_{MPP,STC}}$$

(24) $$r_{2}=\{\begin{array}{cc} {\left(\frac{P_{t+1}}{P_{MPP,STC}}\;\right)}^{2} & if\;\Delta P\geq -\delta_{1}\\ 0 & if\;\Delta P<-\delta_{1} \end{array}$$

(25) $$r_{3}=\{\begin{array}{cc} 0 & if\;0\leq D\leq 1\;\\ -1 & otherwise \end{array}$$

where $\Delta P=P_{t+1}-P_{t}$, $\delta_{1}$ stands for the small number considered as the small area around the maximum power point and used for preventing the, $P_{MPP,STC}$ is the MPP at STC. Here, the reward function includes three components. First, $r_{1}$ is the reward received every time step in a specific episode. This helps the agent to distinguish local and global MPPs, where higher rewards are received if the agent always stays at the global MPPs. Second, based on the value of $r_{2}$, the agent will obtain a positive reward if the power increases, otherwise, zero rewards. Finally, for $r_{3}$, the agent will get a penalty if it is out of the boundary of the duty cycle.

### 3.3. Methodology of the DQN MPPT Control

From the approaches of machine learning, reinforcement learning (RL) methods provide a means for solving optimal control problems when accurate models are unavailable. When dealing with high-dimensional or continuous action domain problems, RL suffers from the problem of inefficient feature representation. What happens when the number of states and actions becomes very large? Additionally, how will we solve complex problems? The answer is solved by the combination of Q learning and Deep learning, named Deep Q Networks (DQN) [39].

The idea is simple: we replace the Q-Table with a Deep Neural Network (Q-Network) which maps environment states to actions of the agent. Generally, DQN used a deep neural network, named as a Q network, to approximate the Q function for the estimation of the return of future rewards. It is denoted as Q(s,a|θ), in which θ is the weights of the neural networks. During the learning process, we use two separate Q networks, including predict Q network with weights θ and target Q network with weights $\theta^{\prime }$ [36,52].

Similarly to supervised learning, in DQN, we can define the loss function as the squared difference between the target and predicted value. Then the network is trained by stochastic gradient descent to minimize the loss function L(θ). Here, it is calculated based on the difference between Q-target and Q-predict as below [36]:

(26) $$L\left(\theta \right)=E_{s,a}\left[{\left(Q_{target}-Q_{predict}\right)}^{2}\right]$$

(27) $$Q_{target}=r+\gamma \underset{a}{max}Q\left(s_{t+1},a_{t+1}|\theta^{\prime }\right)$$

(28) $$Q_{predict}=Q\left(s_{t},a_{t}|\theta \right)$$

During the training, the action is selected based on an ε-greedy policy as given below [53]:

(29) $$a=\{\begin{array}{cc} \underset{a\in A}{argmaxQ(s_{t},a_{t}|\theta )} & if\;b\leq \varepsilon \\ random\left(a_{t}\in A\right) & if\;b>\varepsilon \end{array}$$

where A is the action spaces, b∈[0,1] is a random number, and ε∈[0,1] stands for the exploration rate. When the training starts, the exploitation rate is set to a high value close to 1, and a decay function should be used to lower its value to ensure that the exploitation is conducted as the learning progresses.

**Figure 2:**
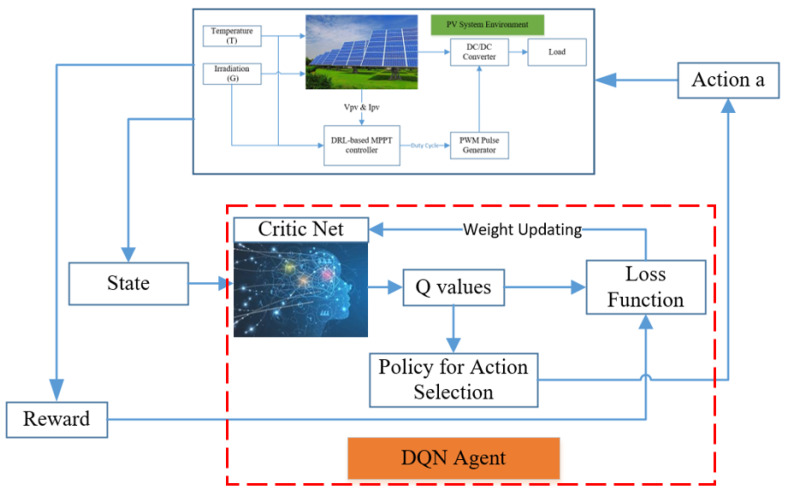
A diagram of the deep Q network (DQN) algorithm.

There are two features that can ensure the learning process is smooth. Firstly, a replay buffer is used for memorizing experiences of the agent behavior. This can help remove the correlation between the agent’s experience and smooth over the changes in the data distribution. Secondly, a mini-batch of transition is randomly sampled from the replay buffer to optimize the mean square error between the prediction and target Q networks. Here, the prediction Q network is updated every time step. On the other hand, the target network is frozen for a period of time steps (C steps in the algorithm) and then the target network weights are updated by copying the weights from the actual Q network. Freezing the target Q network for a while helps stabilize the training process. A diagram of the DQN method is described in Figure 2, while the DQN algorithm can be expressed in Supplementary Figure S6 [34,53].

### 3.4. Methodology of the DDPG MPPT Control

DDPG is an off-policy algorithm. It can deal with continuous action space, so it becomes more applicable for controlling tasks, comparing to DQN which only handles discrete action space [24,42]. On the other hand, it can be considered as the deep Q Learning for continuous action spaces. Different from valued-based methods, policy gradient methods optimize the policy π directly instead of training the value function and choose actions based on it.

In DDPG, four neural networks are used, including a critic Q network ($\theta^{Q}$), an actor deterministic policy network ($\theta^{\mu }$), a target Q network ($\theta^{Q^{\prime }}$), and a target policy network ($\theta^{\mu^{\prime }}$). Both actor net and critic net consist of two neural networks with the same structures, but different weights [50]. The update for critic network is performed by minimizing the loss function as below [24,54]:

(30) $$L\left(\theta^{Q}\right)=Ε\left[{\left(Q_{target}-Q_{predict}\right)}^{2}\right]$$

(31) $$Q_{target}=r+\gamma Q\left(s_{t+1},\mu \left(s_{t+1}|\theta^{\mu^{\prime }}\right)|\theta^{Q^{\prime }}\right)$$

(32) $$Q_{predict}=Q\left(s,a|\theta^{Q}\right)$$

The update of actor is given by minimizing the expected return ($J^{\theta^{\mu }}$) with sampled policy gradient as follows [24]:

(33) $$\nabla_{\theta^{\mu }}\approx Ε\left[\nabla_{\theta^{\mu }}Q\left(s,\mu \left(s|\theta^{\mu }\right)|\theta^{Q}\right)\right]=Ε\left[\nabla_{\mu (s)}Q\left(s,\mu \left(s|\theta^{\mu }\right)|\theta^{Q}\right)\nabla_{\theta^{\mu }}\mu \left(s|\theta^{\mu }\right)\right]$$

**Figure 3:**
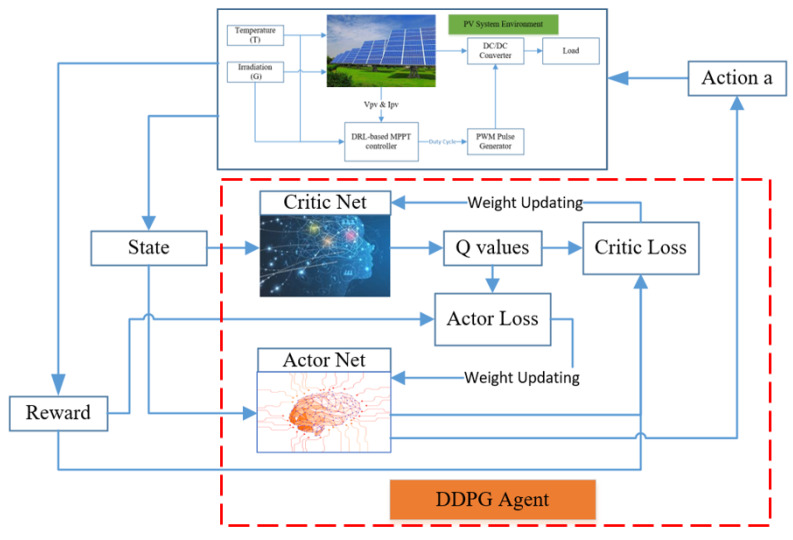
A diagram of the deep deterministic policy gradient (DDPG) algorithm.

Figure 3 shows a diagram of the DDPG method, while Supplementary Figure S7 describes the steps in the DDPG algorithm. As used in DQN and many other RL algorithms, DDPG also uses a replay buffer to sample experience to update neural network parameters. In addition, a mini-batch, randomly sampled from the replay buffer is also used to update the value and policy networks. These help the learning process to be more stable [42]. Compared to DQN, where the target network is updated in a couple of time steps by directly copying the weights from prediction network, in DDPG, the target networks are updated every time step, following the soft update as given below [24,50]:

(34) $$\theta^{Q^{\prime }}\leftarrow \tau \theta^{Q}+(1-\tau )\theta^{Q^{\prime }}$$

(35) $$\theta^{\mu^{\prime }}\leftarrow \tau \theta^{\mu }+(1-\tau )\theta^{\mu^{\prime }}$$

## 4. Simulation and Results

### 4.1. Simulation Set up

The simulation was implemented in Matlab/Simulink through the Reinforcement Learning Toolbox. Based on random initial conditions, including solar irradiation, temperature, and the initial duty cycle, the system was operated with a total time of 0.5 s in an episode and 0.01 s time step. The simulation was conducted within 1000 episodes for both methods. The network layout and number of layers used in this study are recommended by Mathworks. The deep neural networks as shown in Supplementary Figure S8, used to approximate the critic for both DQN and DDPG, have the same setting. It is used for the critic net to approximate the action-value function. Moreover, Supplementary Figure S9 indicated the actor net, which is used to select the action that maximizes the discounted reward. To multiply the input by a weight matrix, a linear function is employed to a fully connected layer. *ReLu* layer is the most popular activation function in deep neural networks, which employs the rectified linear unit activation function. The hyperbolic tangent activation function is used to constraint the output action to the range (−1,1), marked as a *tanh* layer. Then a linear layer is applied to scale up the output from the *tanh* layer to the desired magnitude. In addition, the Adam optimization method is applied for the training of neural networks. The learning rate (α) is defined with the value of 0.001 for both critic networks in two proposed algorithms, while the learning rate of actor network is 0.0001. The action space of DQN is [−0.03, −0.01, −0.005, −0.001, 0, 0.001, 0.005, 0.01, 0.03], while that of DDPG is the range (−0.03,0.03). Moreover, the step of duty cycle used in the P&O method is equal to the value of 0.03. Finally, the other setting parameters are indicated in Table 2. 

### 4.2. Training Results and Performance under STC

**Figure 4:**
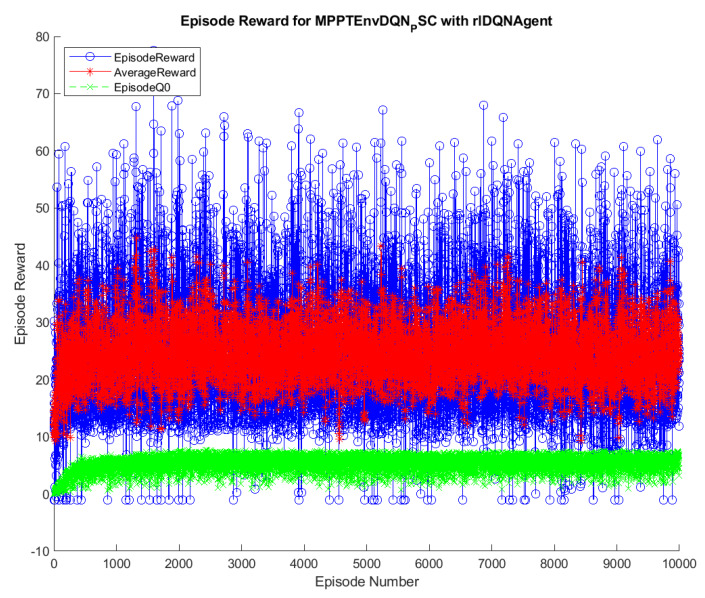
Training process of the DQN method.

**Figure 5:**
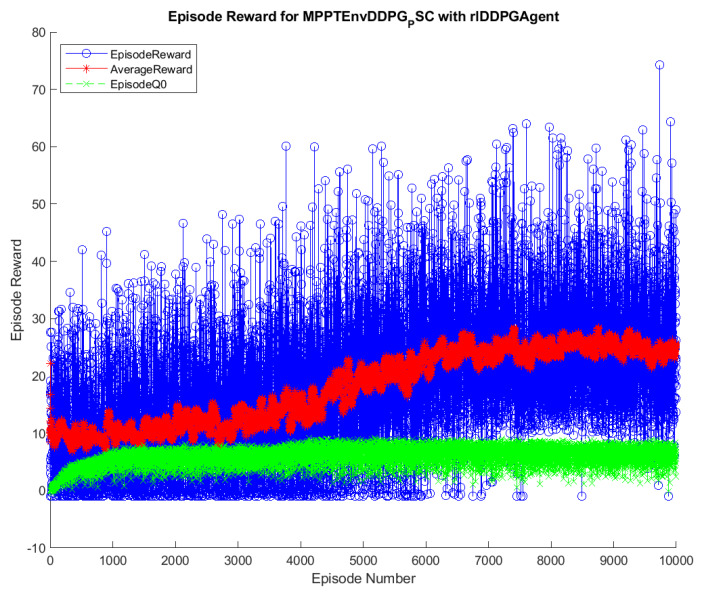
Training process of the DDPG method.

The training results of the DQN and DDPG methods are illustrated in Figure 4 and Figure 5. During the training, the DQN and DDPG agents will save all the interacting information to memory, including state, action, reward, and new-state. In each time step, a mini-batch of the memory is randomly generated for training and updating of the weights of neural networks, respecting each DRL algorithm. As can been seen from the graphs, the blue color indicates the cumulative reward in each episode, marked as Episode Reward. The red one is the average reward during the training process, while the green one is Episode *Q0*. For the agents that have critics, Episode *Q0* shows the estimation of the discounter long-term reward of critics at the beginning of each episode. The training of the DQN method convergences after about 1000 episodes, resulting in the flattened shape of the Average Reward. In contrast, it remains flattened after about 6500 episodes in DDPG. Thus, it can be concluded that the DQN method has less training time than the DDPG method. After being trained, the agents of two methods, including DQN and DDPG, are saved for online control processes. The trained agents are validated through their performance when interacting with the environment. Therefore, various input conditions considered for testing and validation of the proposed methods and the result analysis are described below.

**Figure 6:**
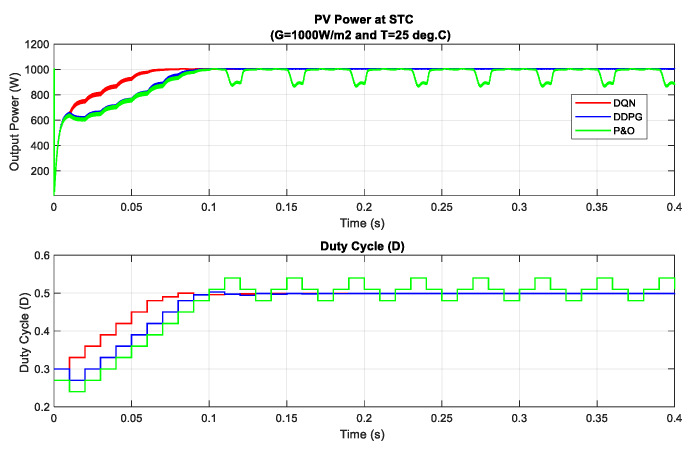
PV power and duty cycle at standard condition.

In the following part, the first set of simulations under standard test condition (STC, *G =* 1000 $W/m^{2}$ and *T =* 15 °C) are carried out to validate the implementation and evaluate the DRL-based MPPT controllers under different operating conditions. In this scenario, the performances of two proposed methods are tested under a standardized operating condition, as well as being compared with traditional MPP tracking method P&O. The simulated results of this scenario are illustrated in Figure 6. As can be seen in the figure, the MPP is tracked after just about 0.07 s for the DQN-based method, while DDPG and P&O almost get the same tracking speed. On the other hand, DQN and DDPG methods are more stable. As the P&O method has oscillation with high magnitude, the two proposed methods perform with a constant duty cycle at about 0.5, which results in the low oscillation of the P–V curve. Based on the results in this scenario, the power tracking efficiency of DQN and DDPG methods increases with the values of about 5.83% and 3.21%, respectively, when compared to that of the P&O method.

### 4.3. Performance under Varying Operating Conditions

**Figure 7:**
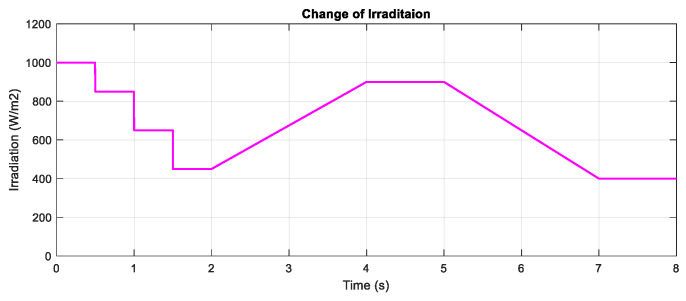
The change of irradiation at T = 25 °C.

**Figure 8:**
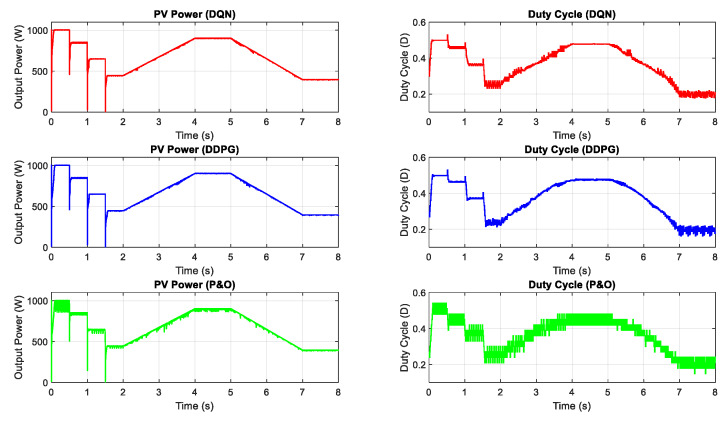
PV power under the change of irradiation.

In this part, the test for the two proposed DRL-based methods under a constant temperature with the change of irradiation is carried out. Figure 7 shows the input condition for this scenario testing, including step change, gradually increasing and decreasing the irradiation. The performances of the three methods are illustrated in Figure 8. All the plots in the left-hand side indicate the PV output power while the plots in the right-hand side describe the control signal of the duty cycle. As can be seen from the graph, the duty cycle of the P&O method changes with higher magnitudes, resulting in higher oscillation around the MPP when compared with the other two methods. Based on the step change of irradiation, the responses of the three methods are almost the same. However, the DQN and DDPG methods perform more stable and smoother, resulting in the thinner PV and duty cycle curves. According to the simulated results in this scenario, the power tracking efficiency of DQN and DDPG increases with the values of about 1.24% and 0.96%, respectively, when compared with the P&O method.

**Figure 9:**
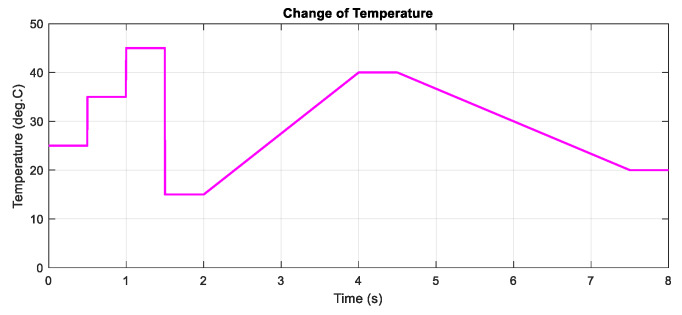
The change of temperature at G = 1000 $W/m^{2}$.

**Figure 10:**
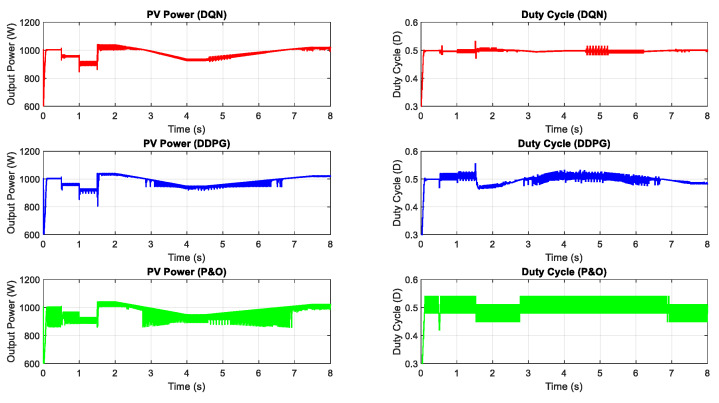
PV power under the change of temperature.

In the following, the two proposed MPP controllers are tested under the change of temperature with a constant input value of the irradiation. Similar to the above scenario, the test is conducted under the step and gradual change of the temperature as can be shown in Figure 9, while Figure 10 describes the PV output power and duty cycle of the three applied methods. Following the graph, it can be concluded that the DQN method has the highest performance with the lowest oscillation, followed by DDPG and P&O methods, resulting in more power tracking. When compared with the P&O method, the efficiency of the DQN method increase by 2.74%, followed by the DDPG method with a value of 2.55%.

**Figure 11:**
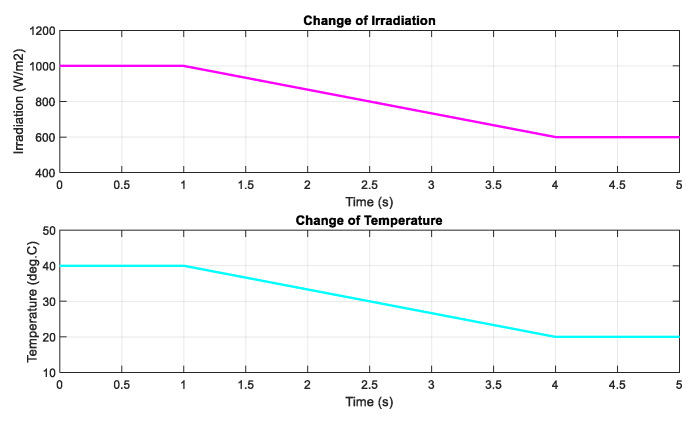
The change of both irradiation and temperature.

**Figure 12:**
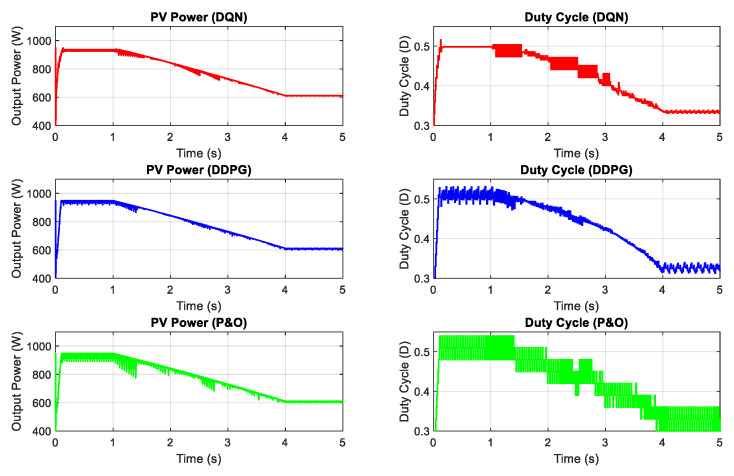
PV power under the change of both irradiation and temperature.

Next, the DRL-based methods are tested under the change of both irradiation and temperature as shown in Figure 11. The operating condition starts with 1000 $W/m^{2}$ and gradually decreases to a value of 600 $W/m^{2}$, while the temperature is set to 40 °C at the beginning and also declines to a value of 20 °C at the end. The performances of the three proposed methods are demonstrated in Figure 12. As shown in the graph, the red line is for the DQN method, while the blue line and green lines indicate the DDPG and P&O, respectively. The graphs on the left-hand side illustrate the output power while the right-hand side graphs show the duty cycle. Under the step change of weather conditions, as shown in the first second and the last second of the graphs, the DQN method has the best performance, resulting in the lowest oscillation of the duty cycle and output power. It is followed by the DDPG and P&O, respectively. However, under the gradual change of both temperature and irradiation, as shown from 1–4 s, DDPG follows the power path better than the other methods, so its duty cycle curve is less oscillating. Thus, the DDPG method has the highest efficiency, followed by DQN and P&O methods. Compared to the P&O method, the power tracking efficiency of the DDPG method increases by 1.62%, while that of the DQN method is just about 1.58%.

### 4.4. Performance under PSC

**Figure 13:**
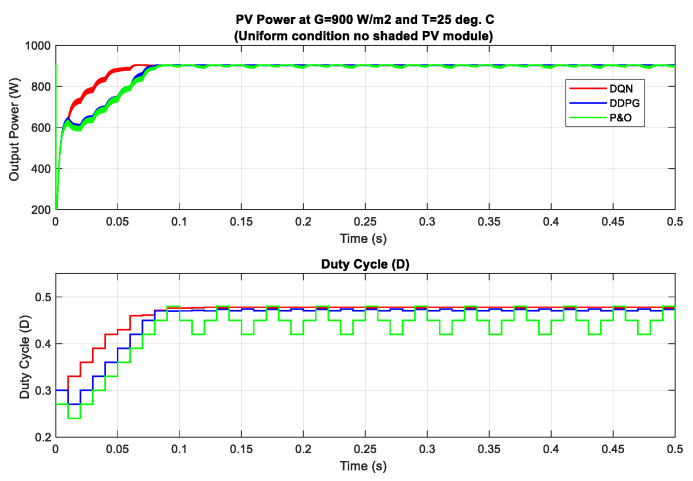
PV power under uniform condition with G = 900 $W/m^{2}$.

In this section, different partial shading conditions are applied for the testing and validation of the proposed methods. There are three PV modules in the PV system and they are connected in series. Firstly, a uniform weather condition at 900 $W/m^{2}$ is applied and the tracking results are displayed in Figure 13. Then, the scenario with one shaded PV module is tested, followed by two shaded PV modules and three shaded PV modules. Under this uniform condition, the theoretical value of the MPP is equal to about 902.8 W. As can be seen from the graph, the DQN method, marked as the red line, has the best tracking speed with the lowest oscillation around the MPP, resulting in the flat duty cycle curve. In contrast, the P&O method, marked as the green line has the poorest performance with the highest oscillation of the duty cycle. When compared to the P&O method in terms of power tracking efficiency, the DQN is higher with a 3.35% increase, while that of the DDPG method is just 3.17%.

**Figure 14:**
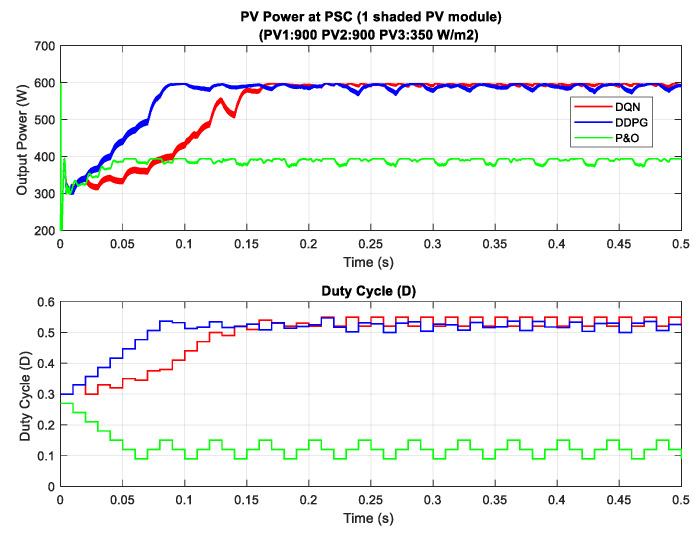
PV power under partial shading condition (PSC) with one shaded PV module.

**Figure 15:**
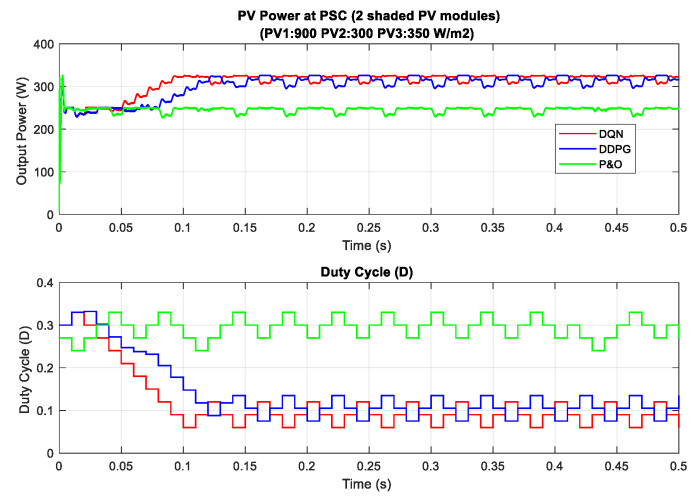
PV power under PSC with two shaded PV modules.

**Figure 16:**
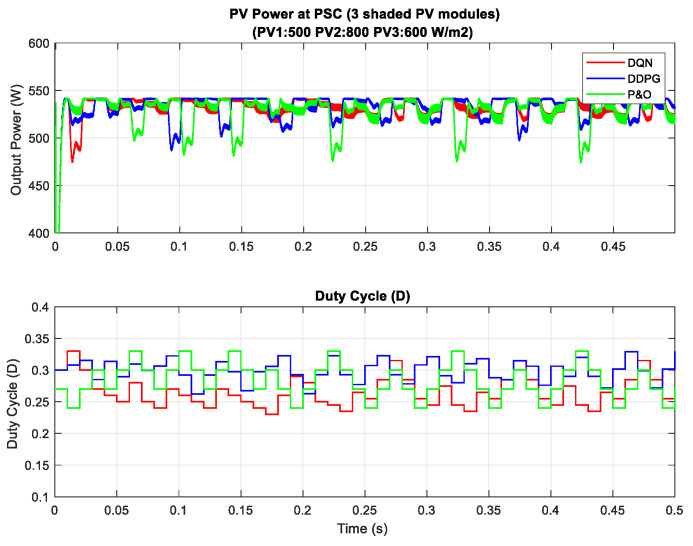
PV power under PSC with three shaded PV modules.

In the scenario with one shaded PV module, the irradiation on one PV module is reduced from 900 to 350 W/m^2^ for testing the response of the proposed MPPT controllers. Additionally, the simulation results are described in Figure 14, in which the upper graph indicates the output power while the lower graph shows the duty cycle. Under this weather condition, the DQN and DDPG can detect the global MPP with a value of around 600 W, marked as the red line and blue line in the figure, respectively. The result reduces by about one third when compared with the uniform state. As can be seen in Figure 14, the green line indicates the result of the P&O method. It can only track the local MPP, resulting in lower power extraction. In this condition, the DDPG method has the highest tracking speed, as well as is the most efficient. Thus, the efficiency of the DDPG method increases by 44.6%, while that of the DQN method is just about 38.3% compared with the P&O method. Next, Figure 15 shows the result of the scenario with two shaded PV modules. In this condition, the values of irradiation on three PV modules are 900, 300, 350 $W/m^{2}$, respectively. On the other hand, the irradiation values on PV modules of the scenario with three shaded PV modules are 500, 800, 600 $W/m^{2}$, respectively, as shown in Figure 16. Similar to the scenario with one shaded PV module, both DQN and DDPG methods are inferior to the P&O method. In Figure 15, compared to the P&O method, the efficiency of DQN and DDPG methods increase by 25.9% and 22.1%, respectively. As shown in Figure 16, these percentages of efficiency are 0.56% and 0.92%. In this case, the P&O method can track the global MPP, however, it is still less efficient than DQN and DDPG methods. It is noted that the DDPG method can extract more power than the DQN based method in the scenarios with one and three shading PV modules.

**Figure 17:**
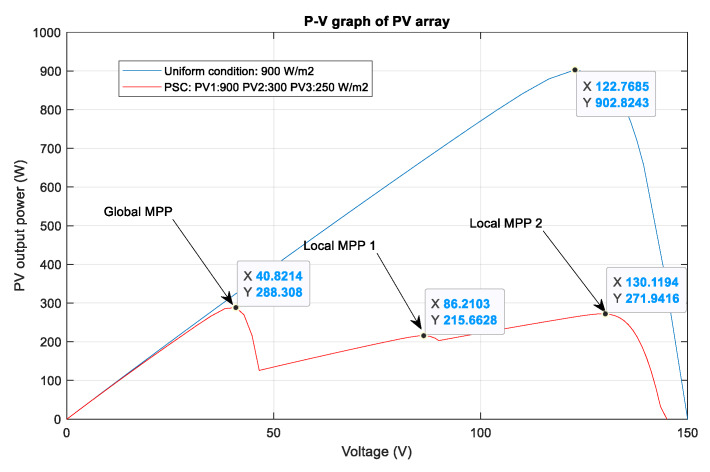
P–V curves of a PV array under uniform and partial shading conditions.

**Figure 18:**
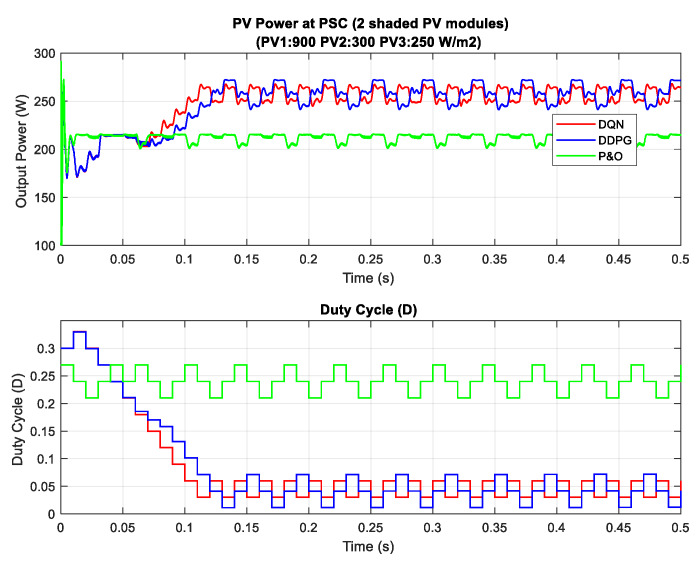
PV power under PSC with a local maximum power point (MPP) tracking.

A summary of the power tracking efficiency under different scenarios simulated in this study is illustrated in Table 3. Most of the time the proposed methods are outstanding in tracking the MPP compared to the P&O method, however, they cannot always obtain global MPP. For example, scenario 8 illustrates the state where the proposed methods cannot track global MPP. Figure 17 describes the P–V curves of the PV array under a uniform condition and a PSC with two shaded PV modules (900, 300, 250 $W/m^{2}$). There are three peaks on the graph, consisting of two local MPPs and one global MPP. In this scenario, the value of the global MPP significantly reduces from about 902.8 W to just around 288.3 W. As can be seen from the tracking results in Figure 18, DQN and DDPG methods can track more power compared to the P&O method, and the powers increase by 17.9% and 15.4%, respectively. However, instead of standing at the global MPP with a value of about 288.3 W, they can only detect the local MPP with a value of around 270 W. Thus, further study should be conducted to improve these potential and efficient methods.

## 5. Conclusions

Besides the development of materials for PV cells to improve the power conversion efficiency, it is essential to develop a new MPPT method which can accurately extract the MPP with high tracking speed under various weather conditions, especially under PSCs. In this study, two robust MPPT controllers based on DRL are proposed, including DQN and DDPG. Both algorithms can handle the problem with continuous state spaces. In which, DQN is applied with discrete action spaces while DDPG can deal with continuous action spaces. The advantage of these two methods is that no prior model of the control system is needed. The controllers will learn how to act after being trained based on the reward received by the continuous interaction with the environment.

Rather than using a look-up table in the RL-based method, DRL uses neural networks to approximate a value function or a policy so that high memory requirement for sizeable discrete state and action spaces could be significantly reduced. Here, the environment is the PV system and refers to the object that the agent is acting on. Here, the agent represents the DRL algorithm, while the action is the perturbation of the duty cycle. It starts by sending a previous state to the agent, which then based on its knowledge, takes action in response to this previous state. Then, the environment responds with a pair of the next state and reward back to the agent. The agent can learn how to take action based on the reward and current state received from the environment. After being trained based on the historical data collected by the direct interaction with the power system, the proposed MPPT methods autonomously regulate the perturbation of the duty cycle to extract the best MPP.

To sum up, compared to the traditional P&O method, the DRL-based MPPT methods applied in this study have a better performance. They can accurately detect the MPP with a significant tracking speed, especially the global MPP under partial shading conditions. In most of the cases, the DQN method overtakes the DDPG method. However, when the partial shading condition happens, the DDPG method slightly outstrips the DQN method. The simulated results show the outstanding performance of the proposed MPPT controllers. However, the limitation of this study is that the proposed method cannot always detect global MPP. Thus, further study will be conducted in the future to improve the tracking ability of DRL-based methods. Furthermore, real-time experiments will be carried out for validation.

## Figures and Tables

**Table 1:** Specifications of American Choice Solar ACS-335-M photovoltaic (PV) module.

Specifications	Value
Maximum Power (W)	334.905
Voltage at MPP (V)	41.5
Current at MPP (A)	8.07
Open circuit voltage, *Voc* (V)	49.9
Short circuit current, *Isc* (A)	9
Temperature coefficient of *Voc* (%/°C)	−0.36
Temperature coefficient of *Isc* (%/°C)	0.09

**Table 2:** Specifications of American Choice Solar ACS-335-M PV module.

Specifications	Value
Replay memory size	$10^{6}$
Batch size	512
Discount factor (γ)	0.9
DQN	
Exploration rate (ε)	1
Decay of exploration rate	0.0001
Exploration rate minimum ($\varepsilon_{min}$)	0.001
DDPG	
Initial variance	0.4
Decay of initial variance	0.0001
Smoothing factor (τ)	0.001

**Table 3:** MPPT tracking efficiency of the proposed methods under various weather conditions compared to P&O.

Scenarios	Weather Conditions	DQN	DDPG
1	Uniform with 1000 $W/m^{2}$	5.83%	3.21%
*2*	*G* changes	1.24%	0.96%
*3*	*T* changes	2.74%	2.55%
4	Both *T* and *G* change	1.62%	1.58%
5	900,900,350 $W/m^{2}$	38.3%	44.6%
6	900,350,300 $W/m^{2}$	25.9%	22.1%
7	500,800,600 $W/m^{2}$	0.56%	0.92%
8	900,300,250 $W/m^{2}$	17.9%	15.4%

## Supplementary Materials

The following are available online at https://www.mdpi.com/1424-8220/20/11/3039/s1. Figure S1. I–V and P–V curves of a PV module under various irradiations; Figure S2. I–V and P–V curves of a PV module under various temperatures; Figure S3. Diagram of three PV modules in series; Figure S4. P–V curve under uniform condition and PSC; Figure S5. Diagram of a typical PV system; Figure S6. DQN algorithm; Figure S7. DDPG algorithm; Figure S8. The structure of critic network in both DQN and DDPG algorithms; Figure S9. The structure of actor network in DDPG algorithm.

## Nomenclature

*G*	Irradiation ($W/m^{2}$)
*T*	Temperature (℃)
*q*	Electronic charge (C)
*k*	Boltzmann’s constant
π	A policy
$V^{\pi }$	Value function
*J*	Objective function
*L*	Loss function
$Q^{\pi }$	Action-value function
*a*	Action
*r*	Reward
*s*	State
θ	Weight matrix
γ	Discount factor
ε	Exploration rate
*I*	Current (A)
*V*	Voltage (V)
*P*	Power (W)
