# Supplementary material for: A Deep Reinforcement Learning-Based MPPT Control for PV Systems under Partial Shading Condition
Source: Sensors (Basel). 2020 May 27;20(11):3039. doi: 10.3390/s20113039 (PMC7308943; doi:10.3390/s20113039)
Supplement: Supplementary file 1 [file sensors-20-03039-s001.pdf]

sensors

Supplementary Materials: A Deep Reinforcement Learning-Based MPPT Control for PV Systems under Partial Shading Condition

Bao Chau Phan [1], Ying-Chih Lai [1,*] and Chin E. Lin [1,2]

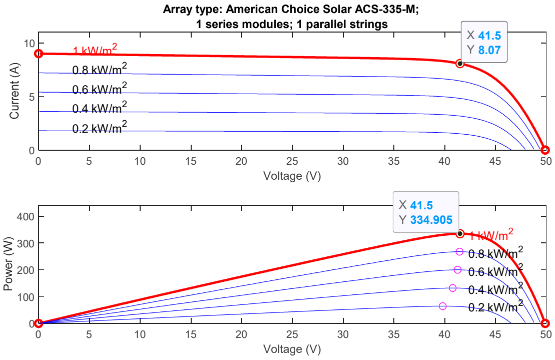

Figure S1. I–V and P–V curves of a PV module under various irradiations.

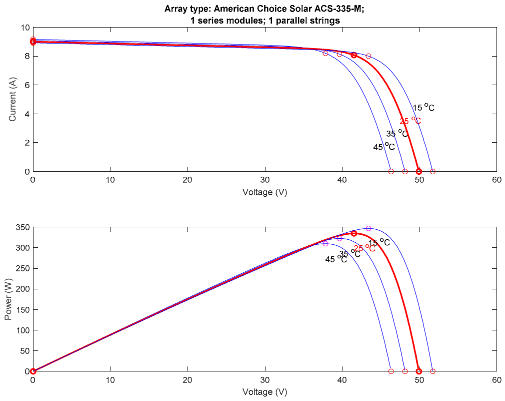

Figure S2. I–V and P–V curves of a PV module under various temperatures.

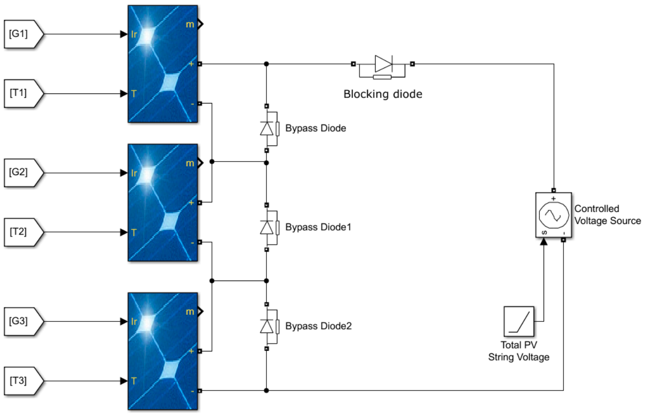

Figure S3. Diagram of 3 PV modules in series.

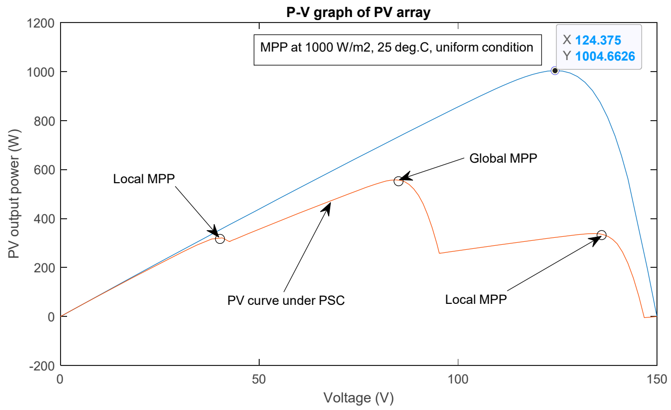

Figure S4. P–V curve under uniform condition and PSC.

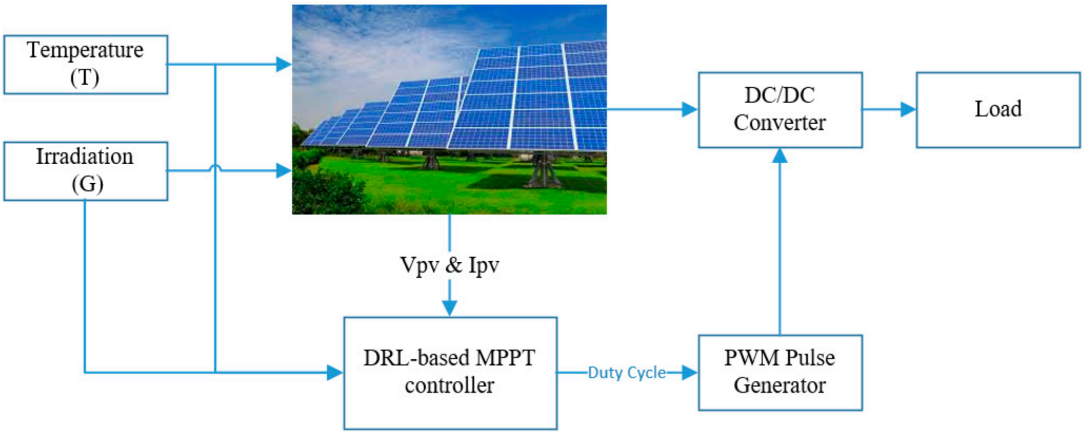

Figure S5. Diagram of a typical PV system.

Algorithm 1: Deep Q Network (DQN)

1. Initialize replay buffer D to a fixed capacity N
2. Randomly initialize critic network $Q(s, a|\theta)$ with weight θ
3. Initialize target networks $Q'(s, a|\theta')$ with weight $\theta' \leftarrow \theta$

for episode = 1:M do
  Initialize state s
  for t = 1: T do
    With the probability smaller of equal to epsilon, select a random action a_t, otherwise select $a_t = argmax(Q(s_t, a_t|\theta))$
    Execute action a_t to get reward r_t and observe the next state s_{t+1}
    Store transition (s_t, a_t, r_t, s_{t+1}) in D
    Sample a random mini-batch from D
    Set

$$y_j = \begin{cases} r_j, if\ episode\ terminates\ at\ the\ step\ k+1 \\ r_j + \gamma \max_a Q(s_{t+1}, a_{t+1}|\theta'), otherwise \end{cases}$$

    Perform gradient descent to minimize the loss function for updating critic network

$$L = \frac{1}{N}\sum_j \left(y_j - Q(s_j, a_j|\theta^Q)\right)^2$$

    Update the weighs of target network every C steps
  end
end

Figure S6. DQN algorithm.

Algorithm 2: Deep Deterministic Policy Gradient (DDPG)

1. Initialize replay buffer D to a fixed capacity N
2. Randomly initialize critic network $Q(s,a|\theta^Q)$ and actor network $\mu(s|\theta^\mu)$ with weight θ^Q and θ^μ, respectively.
3. Initialize target networks Q' and μ' with weight $\theta^{Q'} \leftarrow \theta^Q$ and $\theta^{\mu'} \leftarrow \theta^\mu$, respectively.

for episode = 1:M do
    Initial random process $\mathcal{N}$ for action exploration, and observe initial state s
    for t = 1: T do
        Select action $a_t = \mu_t(s_t|\theta^\mu) + \mathcal{N}$ according to current policy and exploration noise
        Execute action a_t to get reward r_t and observe the next state s_{t+1}
        Store transition (s_t, a_t, r_t, s_{t+1}) in D
        Sample a random mini-batch from D
        Set $y_i = r_i + \gamma Q(s_{t+1}, \mu(s_{t+1}|\theta^{\mu'})|\theta^{Q'}$
        Update critic by minimizing the loss function:

$$L = \frac{1}{N}\sum_i \left(y_i - Q(s_i, a_i|\theta^Q)\right)^2$$

        Update the actor policy using the sampled policy gradient
        Update all the target networks
    end
end

Figure S7. DDPG algorithm.

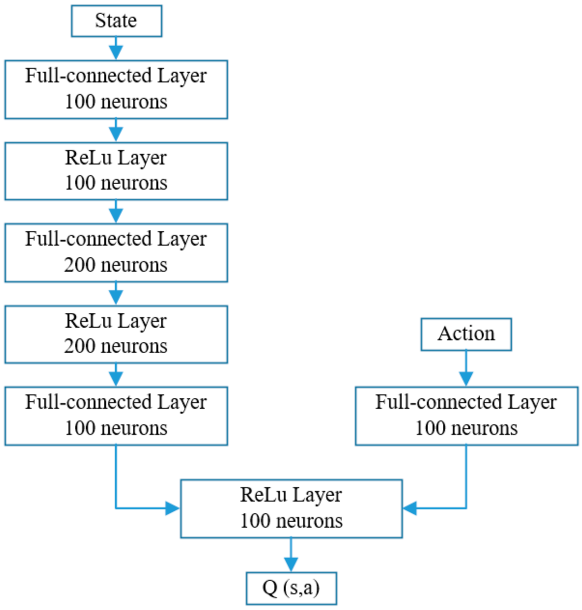

Figure S8. The structure of critic network in both DQN and DDPG algorithms.

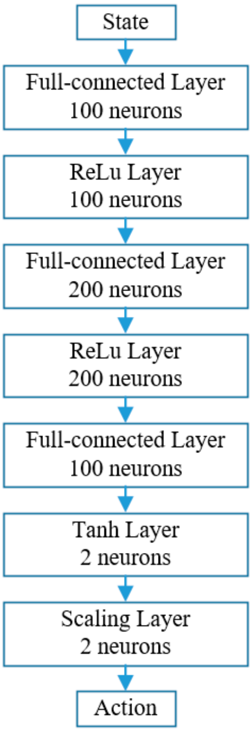

Figure S9. The structure of actor network in DDPG algorithm.

© 2020 by the authors. Licensee MDPI, Basel, Switzerland. This article is an open access article distributed under the terms and conditions of the Creative Commons Attribution (CC BY) license (http://creativecommons.org/licenses/by/4.0/).
